# Reconfigurable absorptive and polarization conversion metasurface consistent for wide angles of incidence

**DOI:** 10.1038/s41598-023-45351-5

**Published:** 2023-10-24

**Authors:** Ahsaan Gul Hassan, Muhammad Sumaid, Fahad Ahmed, Nosherwan Shoaib, Qammer H. Abbasi, Symeon Nikolaou

**Affiliations:** 1 School of Electrical Engineering and Computer Science (SEECS), National University of Sciences and Technology (NUST), Islamabad, 44000 Pakistan; 2 School of Aviation Engineering and Technology, Superior University, Lahore, Pakistan; 3 INRS-EMT, University de Quebec, Quebec, Canada; 4 James Watt School of Engineering University of Glasgow, Glasgow, G12 8QQ UK; 5 Frederick Research Center and the Department of Electrical Computer Engineering and Informatics, Frederick University, 1036 Nicosia, Cyprus

**Keywords:** Electrical and electronic engineering, Electronic devices

## Abstract

In this paper, a single-layer reconfigurable reflective metasurface is presented. The proposed metasurface operates at 5.4 GHz and can achieve either absorption or cross-polarization conversion corresponding at two different diode biasing states. The reflective metasurface acts as an absorber for an incident wave when the diodes are forward-biased. Similarly, it changes the polarization state of the reflected wave for a linearly polarized incident wave when the diodes are reverse-biased. The proposed structure maintains the aforementioned performance characteristics for oblique incidence, up to 60° compared to the perpendicular incidence. The proposed metasurface can achieve linear to linear polarization conversion with polarization conversion ratio (PCR) > 95% and absorption, with absorption ratio (AR) > 80% in the same frequency band just by reconfiguring the state of the PIN diodes.

## Introduction

Metasurfaces are two-dimensional periodic artificially engineered structures that can potentially control and alter the amplitude, phase, and polarization of the incident electromagnetic (EM) waves. The versatile nature of metasurfaces enables a wide range of applications such as polarization conversion, absorption, hologram, antenna gain enhancement, beam steering, flat lenses, anomalous reflection, and asymmetrical transmission^1^. Among these metasurfaces, the metasurface-based absorbers and cross-polarization converters have attracted the attention of researchers due to their potential application in RCS reduction and polarization control^2–9^.

In this perspective, various frequency, and polarization reconfigurable metasurfaces have been investigated^10^. In the case of frequency re-configurability, the operational frequency of the designed surface can be changed by varying the capacitance of the varactor diode. Zhao, Jie et al. reported an absorber in reflection mode, based on two coupled ring resonators connected with a varactor diode^11^. The absorption is electrically tuned by changing the capacitance of the varactor diode in the C-band. In^12^, an integrated circuit (IC) is used to make an absorptive metasurface reconfigurable. The loading elements (LEs) of the IC are integrated with the planner metasurface, then the capacitance of LEs is varied to tune the frequency in S-band. Fei, Peng et al. also reported a frequency reconfigurable chiral metasurface for polarization conversion. The operational frequency can be tuned from 4.17 to 6.73 GHz by switching the PIN diode from OFF to ON state^13^.

**Figure 1:**
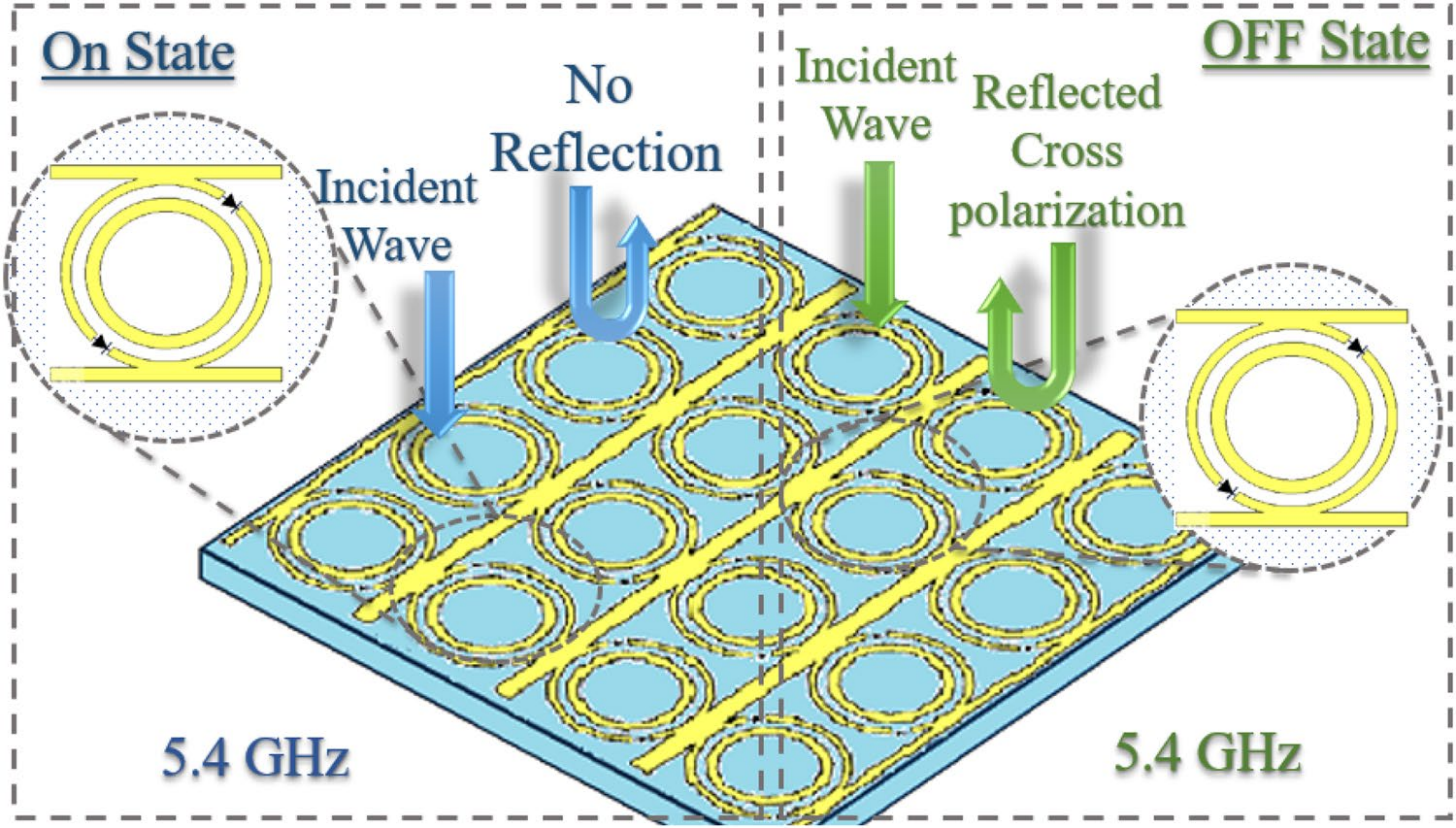
The concept of reconfigurable absorptive and polarization conversion in ON and OFF states of diode.

On the other hand, in the case of polarization reconfigurable metasurfaces, the nature of polarization of the incident EM wave can be changed without changing the frequency by switching the state of the PIN diode^14^. Li, Wei et al. presented a reconfigurable polarization conversion metasurface that can perform linear to circular polarization conversion when the diode is forward biased, and on the other hand, it simply reflects the wave without any polarization transformation when the diode is reverse biased^15^. Gao, Xi et al. incorporated varactor diode in butterfly patch reconfigurable metasurface for performing polarization conversion. The integrated varactor diodes can perform linear-to-linear polarization conversion at zero bias and linear-to-circular polarization conversion at a bias voltage of − 19 V^16^. Another broadband polarization reconfigurable metasurface is reported which can perform linear-to-linear polarization, and linear-to-circular polarization conversion in the reverse and forward bias state of a PIN diode in the frequency range of 10.5 to 13 GHz, respectively^17^. Recently, a wide band polarization conversion reconfigurable metasurface for 5G application was presented. It can perform linear-to-linear polarization conversion in the frequency range of 30 to 42 GHz, and linear-to-circular polarization conversion in the frequency range of 19 to 40 GHz for the ON and OFF states of the diodes, respectively^18^. Even though an ample amount of research work was carried out on reflective AMS, according to the best of the authors’ knowledge, no single-layer switchable reconfigurable metasurface has been reported that can switch between polarization conversion and absorption functionality according to the two bias states of the used diodes in a fixed frequency band. In this letter, an active single-layer metasurface consisting of an array of dual ring-shaped resonators with parallel stubs is presented. The proposed metasurface can achieve linear to linear polarization conversion with polarization conversion ratio (PCR) > 95% and the absorption of the incident wave, with absorption ratio (AR) > 80% in the same frequency band by switching the state of the PIN diodes from OFF to ON, respectively. The proposed design is also angularly stable for angles of incidence up to 60°. The concept of the proposed metasurface along with its functionality is depicted in Fig. 1.

## Unit cell design

### Geometrical configuration

**Figure 2:**
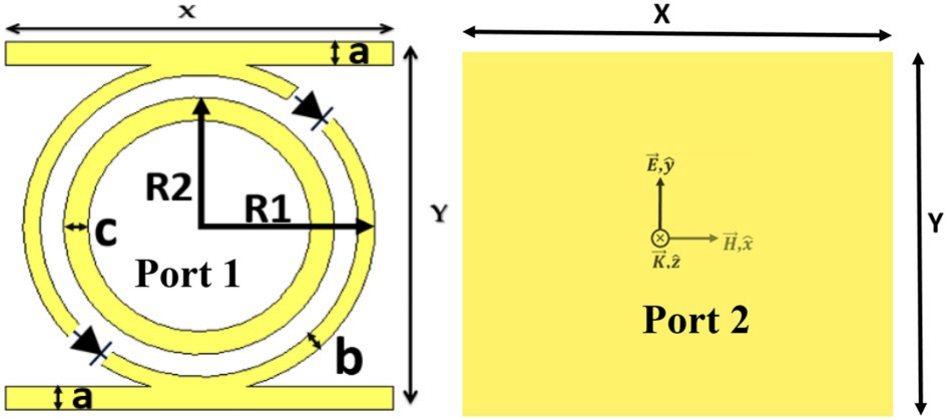
Unit cell design structure: (Port 1) Front structure (Port 2) Ground reflector.

The proposed unit cell structure (see Fig. 2) consists of a ring and a split ring resonator backed with two parallel stubs. The PIN diodes (BAR64-02 V) are integrated into the two gaps of the split ring resonator along a direction that forms 45 degrees angle with x-axis as shown in Fig. 2. The top and ground layers of the designed structure are separated by a 3.2 mm FR-4 substrate (${\varepsilon }_{\mathrm{r}}=4.2$ and loss tangent of 0.025). The optimized parameters of the proposed design are: a = 1, b = 0.7, c = 1, R_1_ = 7.27, R_2_ = 5.6, and X = Y = 16 (all in mm).

### Theoretical background

The fundamental concepts of absorptive metasurfaces are based on the multiple reflection theory and the impedance matching theory^19^. In the case of multiple reflection phenomena^20^, the incoming EM wave passes through the designed structure and substrate and is then reflected from the ground layer. After passing through the substrate layer, the reflected wave is again reflected back, and the process continues until the wave diminishes. The metasurface acts as a coupled system where anti-parallel currents are induced between the top and ground layers to create magnetic resonance. The partially reflective surface of the metasurface can modify the complex reflection and transmission coefficients, whereas the perfect electric ground plane offers the phase shift of 180°. The mechanism of multiple reflections and transmissions inside a dielectric substrate can be written as a superposition of the reflection modes (Eq. 1).

1 $$\mathrm{R}\left(\upomega \right)= {\mathrm{R}}_{12}\left(\upomega \right)\times \left(\frac{{\mathrm{T}}_{12}\left(\upomega \right) {\mathrm{T}}_{21}\left(\upomega \right) {\mathrm{e}}^{-2\mathrm{i\beta }}}{1+ {\Gamma }_{21}\left(\upomega \right) {\mathrm{e}}^{-2\mathrm{i\beta }}}\right).$$

In the case of the impedance matching mechanism^21^, the impedance of the designed structure is matched with the impedance of air, to absorb any incoming EM waves. Such metasurfaces are based on magnetic resonant response which causes anti-parallel surface currents on the cell plane. This phenomenon can be well explained through the Fresnel reflectivity formula (Eq. 2)^2^.

2 $$\mathrm{R}\left(\upomega \right)={|\Gamma |}^{2}= {\left|\frac{\mathrm{xcos\theta }- \sqrt{{\mathrm{n}}^{2}-\mathrm{cos\theta }}}{\mathrm{xcos\theta }+ \sqrt{{\mathrm{n}}^{2}-\mathrm{cos\theta }}} \right|}^{2},$$

where ${\mathrm{Z}}_{\mathrm{o}}$ is the intrinsic impedance of free space and Z(ω) refers to the metasurface impedance. For high absorption, ${\mathrm{Z}}_{\mathrm{o}}$ should be equal to Z(ω) to eliminate any reflections (i.e. S_11_ = 0). The absorption ratio can be expressed as follows:

3 $$\mathrm{A}\left(\upomega \right)= 1 -\Gamma {\left(\upomega \right)}_{\mathrm{cross}}-\Gamma {\left(\upomega \right)}_{\mathrm{co}}-\mathrm{ T}{\left(\upomega \right)}_{\mathrm{cross}}-\mathrm{ T}{\left(\upomega \right)}_{\mathrm{co}}.$$

The Eq. (3) can also be expressed as^22^:

4 $$\mathrm{A}(\upomega ) = 1 - {|{R}_{xx/yy}|}^{2} - {|{R}_{xy/yx}|}^{2}.$$

The proposed metasurface is fully grounded with copper, therefore, the transmission coefficient is zero. Hence Eq. (3) can be written as:

5 $$\mathrm{A}(\upomega ) = 1 -\Gamma (\upomega )= 1 - {|{\mathrm{S}}_{11}|}^{2} = 1- { \left|\frac{\mathrm{Z}\left(\upomega \right)-{\mathrm{Z}}_{\mathrm{o}}(\upomega )}{\mathrm{Z}\left(\upomega \right)+{\mathrm{Z}}_{\mathrm{o}}(\upomega )} \right|}^{2}.$$

The underlying physics of linear-to-linear polarization conversion metasurfaces is explained through the Faraday effect^23^. The incident EM wave undergoes a phase shift of 90° and the horizontally polarized wave is transformed into the vertically polarized wave and vice versa. If the x-polarized wave ($$\mathop{E}\limits^{\rightharpoonup} _{i} = \hat{x} E_{o} e^{ - jkz}$$) is incident on the cross polarizer, then the reflected wave can be written as $$\mathop{E}\limits^{\rightharpoonup} _{r} = \mathop{E}\limits^{\rightharpoonup} _{i} . e^{{{\raise0.7ex\hbox{${j\pi }$} \!\mathord{\left/ {\vphantom {{j\pi } 2}}\right.\kern-0pt} \!\lower0.7ex\hbox{$2$}}}}$$. To evaluate the performance of the linear-to-linear polarization conversion metasurface, the polarization conversion ratio (PCR) is defined as follows:

6 $${PCR}_{y}=\frac{{R}_{yx}^{2}}{{R}_{yx}^{2}+{R}_{xx}^{2}} .$$

### Design evolution

To achieve the desired characteristics, multiple steps were taken into consideration to achieve nearly perfect absorption and polarization conversion at the same frequency by reconfiguring the state of the PIN diodes incorporated in the unit cells. Firstly, the absorption and polarization conversion is achieved by assuming the ideal states of the diodes (short circuit for the forward bias and open circuit for the reverse bias). When the diodes are in reverse bias, the design will be ideally (assuming an open circuit) symmetric along the x and y axes, while asymmetric along the u and v axes which enables the metasurface for cross-polarization conversion^1^. In the case of forward biasing, the design will be ideally (assuming a short circuit) symmetric along the x, y, u, and v axes, that enables it for absorption^4^. To understand the absorption of the ring resonator, consideration can be directed toward bound states in the continuum (BICs). These BICs represent distinct states that remain localized indefinitely, despite residing within the radiation continuum^24^.

**Figure 3:**
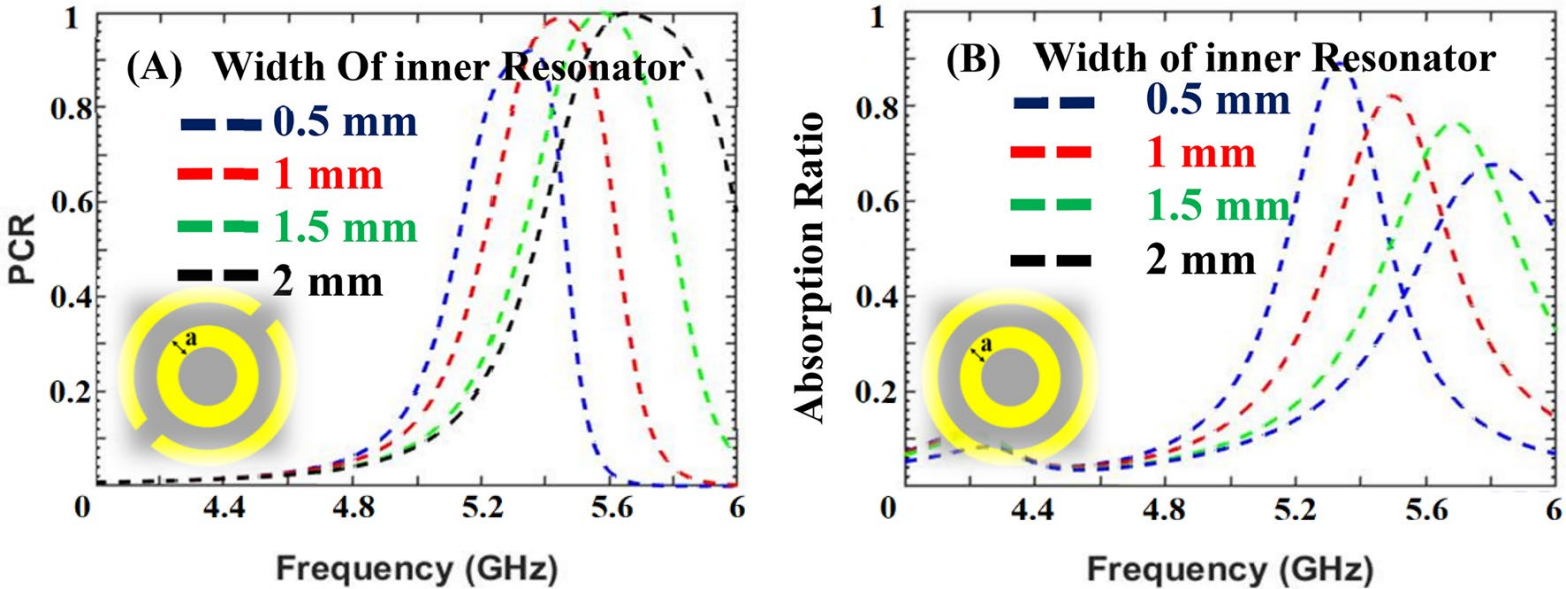
Trade-off due to width of inner resonator: (**A**) Polarization conversion ratio; (**B**) Absorption ratio.

**Figure 4:**
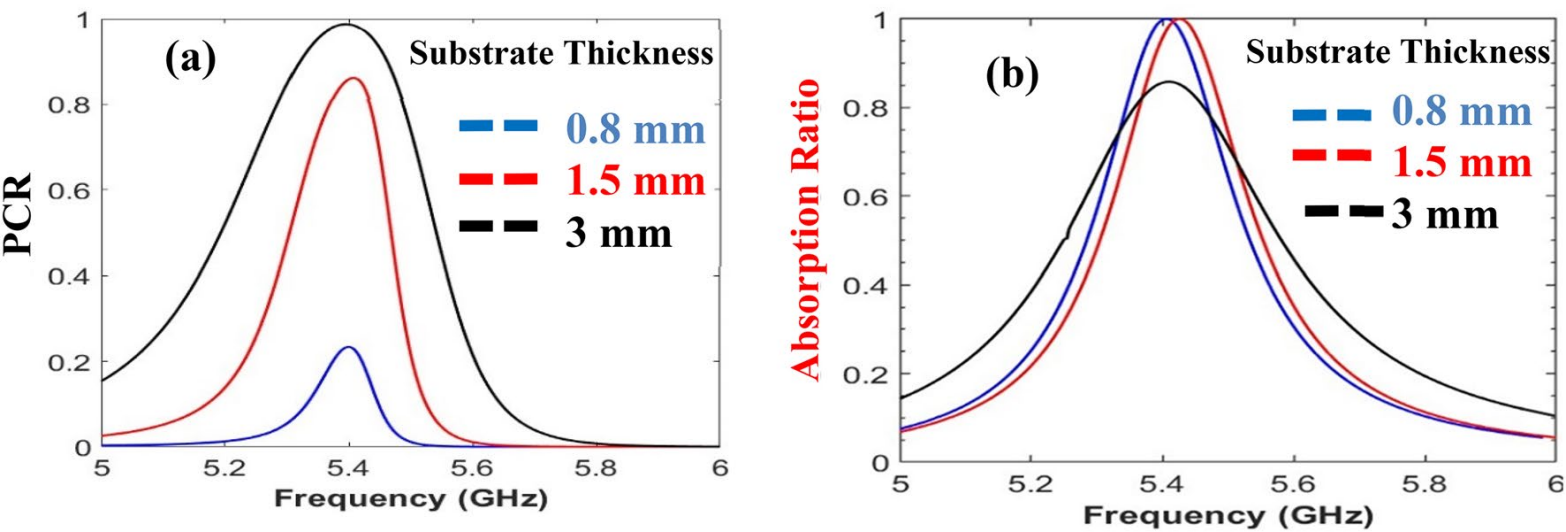
Trade-off due to substrate thickness between (**a**) polarization conversion ratio; (**b**) absorption.

**Figure 5:**
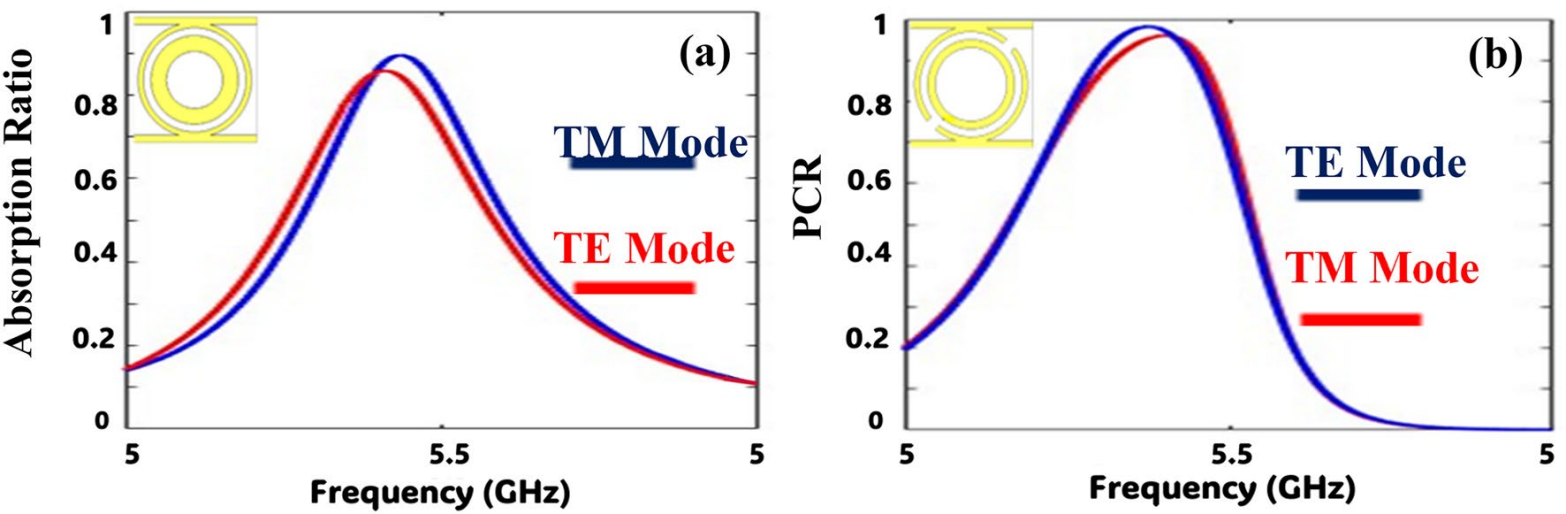
Ideal designed structures with feed-line stubs: (**a**) absorption; (**b**) polarization conversion ratio.

The trade-off between absorption and polarization conversion is analyzed and the final parameters are selected accordingly. The changes in the thickness of the substrate and the width of the inner resonator leads to a modification in the value of the capacitance, thus, causing changes in the surface impedance of the designed structure and, consequently, affecting the functionality of the design^5^. It is observed that by increasing the width of the inner ring resonator and the thickness of the substrate the PCR increases, while the AR decreases as depicted in Figs. 3 and 4. The optimized unit cell design is backed with feed line stubs and integrated with the PIN diode. All diodes throughout the designed periodic sheet are placed in the same manner. When the diodes are in the OFF state high resistance is caused, which isolates the split ring resonators. The isolation between the split ring resonators creates C2 symmetry which enables linear-to-linear polarization conversion^23^. In the ON state of the diodes, low resistance is caused which allows EM waves to pass through the split ring resonator and offers C4 symmetry. The C4 (90° rotated) symmetric nature of the metasurface provides absorption^25^. The AR and the PCR for the designed metasurface are greater than 80% and 95% respectively, as can be verified from Fig. 5.

## Metasurface performance

### Reflection coefficient

**Figure 6:**
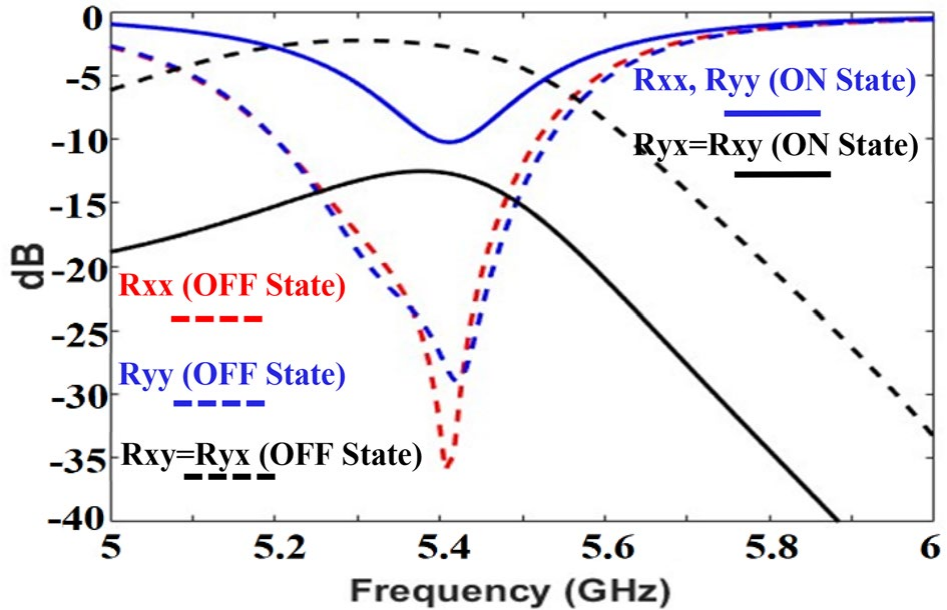
S-parameters of the proposed design in the ON and OFF states of the diode.

The proposed active metasurface was simulated in CST 2021 Microwave Studio® and periodic boundary conditions were applied to the unit cell. The selected diodes were modeled as lumped (RC) elements in ON and OFF states. The transmission value is zero because of the reflective nature of the metasurface (the bottom is a perfect electric conductor). The co- (Rxx, Ryy) and cross- (Rxy, Ryx) components for reflection at port 1 are shown in Fig. 6. To achieve absorption (> 80%) Rxx, Ryy, Rxy, and Ryx should be less than − 10 dB. On the other hand, for the functionality of cross-polarization conversion (PCR > 90%), the co-components (Rxx and Ryy) should be less than − 10 dB, and cross-components (Rxy and Ryx) must be closer to 0 dB. It can be noted from Fig. 6 that the criteria for the absorption are achieved at 5.4 GHz for the ON state of the diodes, whereas the polarization conversion criteria are met at the same resonant frequency, for the OFF state of the diodes.

### Absorption and polarization conversion

The absorption is calculated from Eq. (4) at port 1 for the ON state of the diodes. The AR is greater than 80% at 5.4 GHz for linearly polarized (TE/TM) and circularly polarized incident waves. Similarly, the PCR is calculated from Eq. (6) at port 1 for the OFF state of the diode. In the OFF state of the diodes, the split ring resonators are isolated, and the incident x-polarized wave is converted into the y-polarized wave and vice versa. The PCR of the proposed metasurface is higher than 95% at 5.4 GHz. In the ON state of the diode, it provides very low resistance between the split ring resonators (offers C4 symmetry), and the incident EM wave is absorbed by the structure. The AR of the proposed metasurface is greater than 80% at 5.4 GHz.

### Current distribution analysis

**Figure 7:**
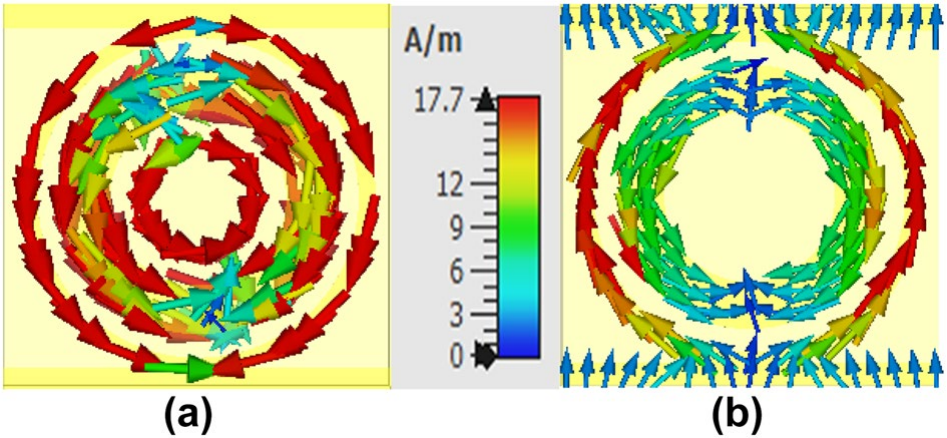
Surface current: (**a**) off state of diode: (**b**) on state of diode.

The phenomena of absorption and cross-polarization conversion can be understood with the aid of surface current analysis. The surface current distribution in the two bias states of the diodes is shown in Fig. 7. For polarization conversion the orthogonal alignment of currents changes the phases of the electric and magnetic components^26^. On the other hand, in the case of absorption, the magnetic field produced within the slab due to opposite current flow and the underlying physics can be explained through the impedance matching theory^19^.

### Angular stability and LC circuit modeling

**Figure 8:**
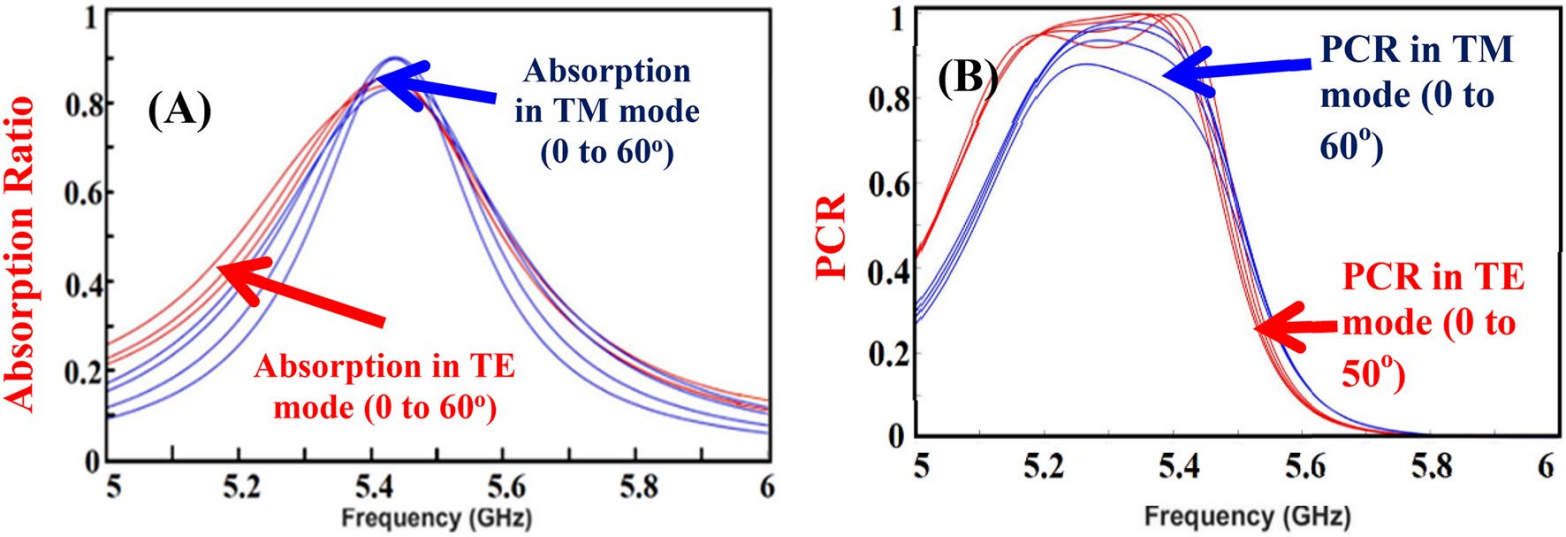
Oblique incidence performance for (**A**) Forward bias and (**B**) Reverse bias polarization.

**Figure 9:**
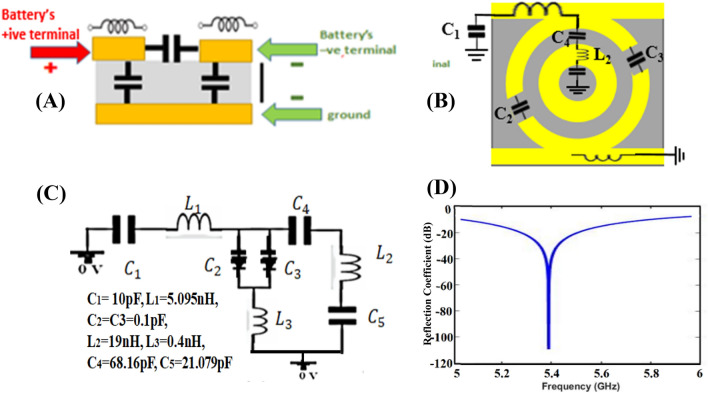
Equivalent circuit modeling and verification of the analogous transmission line model simulated ADS software.

Angular stability is the key parameter to define the performance of the designed metasurface i.e. a similar response to different oblique angles of incidence. For most practical applications, angular stability is required^27^. Generally, it depends on the geometry of the unit cell, the size of the unit cell, and the dielectric substrate thickness^28^. A simple structure is proposed, and the electrical length and thickness of the unit cells are also kept small to achieve high angular stability. The reconfigurability of the designed surface remains almost the same for wide angles of incidence up to 60°. The angular stability of the designed metasurface is shown in Fig. 8. To define the resonant frequency of the proposed design, its LC circuit is designed and analyzed in ADS. The unit cell is analyzed using the transmission line theory analogous. For this model, the port is placed on the top of the structure, and the terminals of the port are connected to the top and bottom of the structure. Starting from the top of the structure, the conductive length of the structure is modeled as an inductor, and the discontinuity between two conductive segments is modeled as capacitance. Additional capacitors are used to model the capacitance between the conductive segments of the top layer and the bottom ground plane. Figure 9 shows the equivalent circuit modeling and verification of the analogous model.

## Experimental analysis

**Figure 10:**
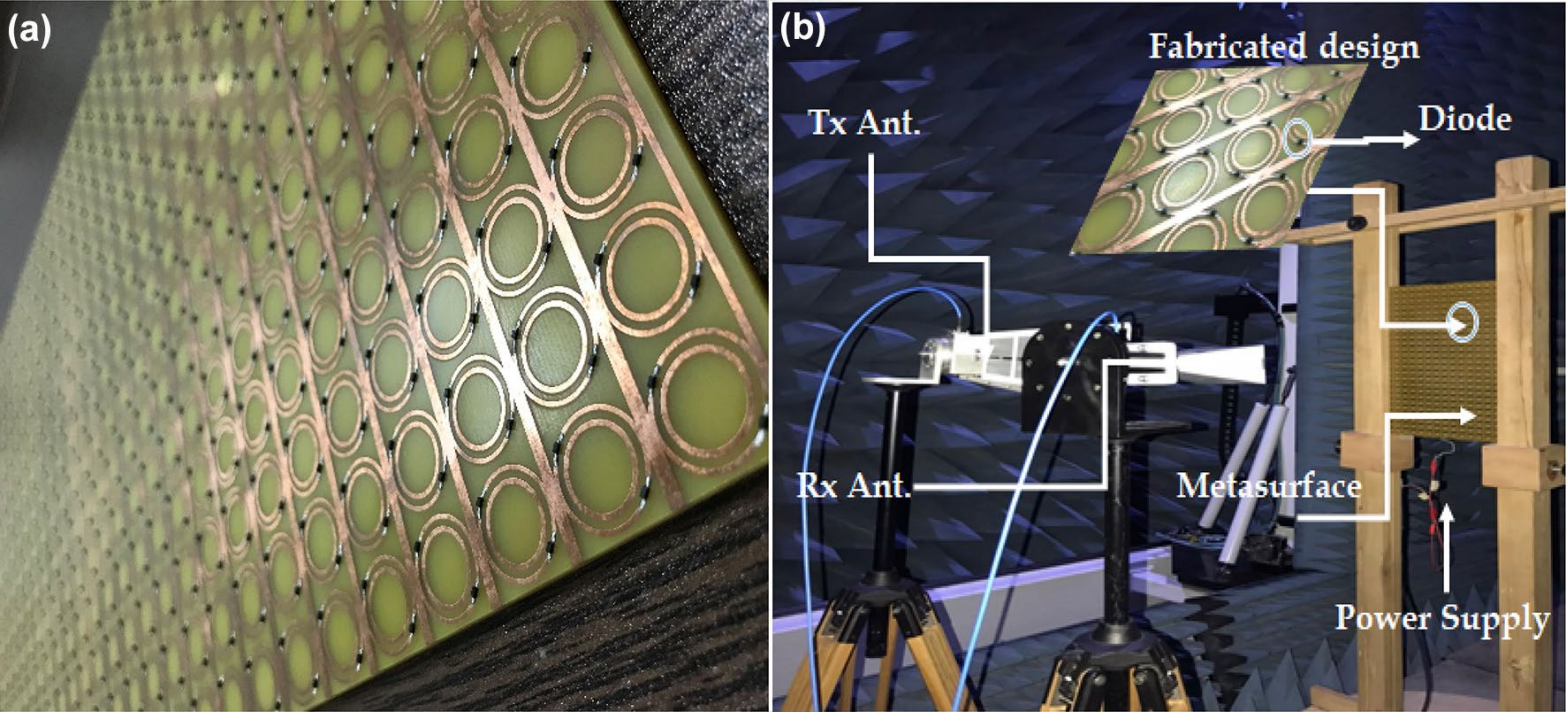
Prototype of designed reconfigurable metasurface: (**a**) Fabricated surface; (**b**) Experimental setup.

**Figure 11:**
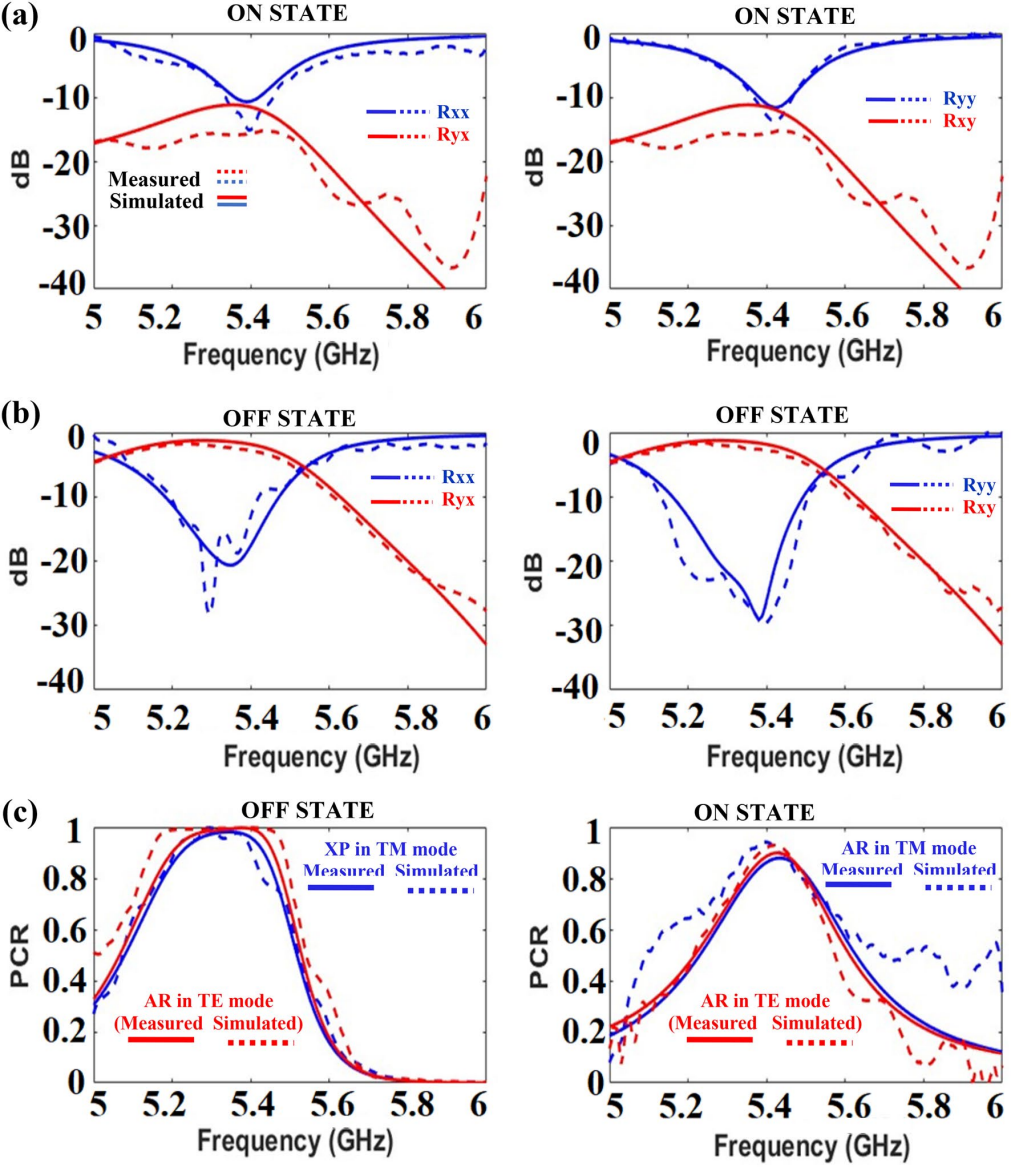
Measured vs. simulated: (**a**) on state of diode; (**b**) OFF state of diode (**c**) absorption and polarization.

To validate the simulated results, the designed metasurface is fabricated on an FR-4 substrate of dimensions 256 × 400 mm^2^. An array of 16 × 25-unit cells was fabricated by a chemical etching process. The fabricated metasurface and the experimental setup in an anechoic chamber are illustrated in Fig. 10. Two horn antennas (4 to 8 GHz) were used to generate the incident and receive the reflected EM waves^29^. Since the designed surface is reflective, both antennas were placed on the front side (to transmit and receive) of the surface. The experimental setup is shown in Fig. 10. The designed metasurface was tested at different angles of incidence as the distance placed the metasurface in the Fresnel region^30^. The upper side of the surface is connected to the positive terminal and the lower side to the negative terminal of the supplying battery. To achieve the metasurface absorptive characteristics, the DC voltage set the diodes in the ON state, while for achieving the alternative behavior with polarization conversion, the DC supply was just disconnected. In both biasing states of the diodes, the reflection coefficients were measured and compared with the simulated results. The reflected parameters, shown in Fig. 11, present a good agreement between measured and simulated results. A comparison table with relevant state-of-the-art metasurfaces is shown in Table 1. It is evident that the proposed metasurface achieves alternative functionality (absorption or polarization conversion at a fixed controlled frequency) compared to the published work for a significantly wider angle of oblique incidence (60°).

**Table 1:** Comparison with other active surfaces.

Reference	Active	Reflective	Functionality			Angular stability
			Absorption	Polarization conversion		
				Cross	Linear to circular	
^11^	✓	✓	✓	x	x	50°
^12^	✓	✓	✓	x	x	N/G
^13^	✓	x	x	✓	x	N/G
^14^	✓	✓	x	✓	✓	N/G
^15^	✓	✓	x	✓	✓	N/G
^16^	✓	✓	x	✓	✓	30°
^17^	✓	✓	x	✓	✓	N/G
^18^	✓	✓	x	✓	✓	30°
This work	✓	✓	✓	✓	x	60°

## Conclusion

In this paper, a novel single-layer reconfigurable metasurface for radar cross-section (RCS) reduction and polarization conversion applications is presented. The metasurface consists of split ring resonators, with embedded PIN diodes that allow for reconfigurability. In the ON state of the diodes, the proposed metasurface can perform absorption (> 80%) at 5.4 GHz whereas in the OFF state, cross-polarization conversion (PCR > 90%) at the same frequency is performed. In addition, the presented metasurface achieved significant angular stability characteristics for wide angles of oblique incidence, up to 60°.

## Data Availability

The datasets used and analyzed during the current study are available from the corresponding author upon reasonable request.
